# Comprehensive analysis of performance, physiological, and perceptual responses during an entire sprint cross-country skiing competition

**DOI:** 10.1007/s00421-023-05326-w

**Published:** 2023-10-07

**Authors:** Rune Kjøsen Talsnes, Tore Berdal, Jan-Magnus Brattebø, Trine Seeberg, Thomas Losnegard, Jan Kocbach, Øyvind Sandbakk

**Affiliations:** 1 Centre for Elite Sports Research, Department of Neuromedicine and Movement Science, Norwegian University of Science and Technology, Trondheim, Norway; 2 Department of Physical Performance, Norwegian School of Sport Sciences, Oslo, Norway

**Keywords:** Competitive demands, Endurance sports, Rating of perceived exertion, Skating, Time trial, XC skiing

## Abstract

**Purpose:**

To investigate performance, physiological, and perceptual responses of an entire sprint cross-country skiing competition in the skating style.

**Methods:**

Eighteen national-level male junior skiers participated in a simulated competition comprising an individual time trial (TT), followed by three heats (quarterfinals [QF], semifinals [SF], and final [F]). Participants’ heart rate (HR) was continuously monitored while perceived readiness (RED, 1–10), rating of perceived exertion (RPE, 6–20), and blood lactate [La-] were assessed at standardized time points.

**Results:**

The total duration and distance covered were 03:30 ± 00:06 h and 25.2 ± 2.9 km, respectively. The participants spent 02:19 ± 00:27 h > 60% of their maximal HR (HR_max_) and 00:16 ± 00:04 h > 85% of HR_max_. Average HR decreased from TT to F (89.3 ± 2.0% vs. 86.9 ± 3.0% of HR_max_, *P* < 0.01). [La-] levels were highest before (4.6 ± 2.0 vs. 2.9 ± 1.2, 3.2 ± 2.0 and 2.5 ± 1.3 mmol·L^−1^, all P < 0.01) and after (10.8 ± 1.4 vs. 9.8 ± 1.6, 9.1 ± 1.8 and 8.7 ± 1.7 mmol·L^−1^, all *P* < 0.05) F compared to TT, QF, and SF, respectively. RED was lowest before F compared to TT, QF, and SF (6.6 ± 1.4 vs. 7.9 ± 1.1, 7.6 ± 1.1, and 7.4 ± 1.4, respectively, all *P* < 0.05) while RPE was highest after TT compared to QF, SF, and F (17.8 ± 0.9 vs. 15.1 ± 2.0, 16.5 ± 1.2 and 16.6 ± 1.8, respectively, all *P* < 0.01). The six best-performing skiers demonstrated higher RED before F (7.2 ± 0.9 vs. 5.3 ± 1.2, *P* < 0.05) and higher [La-] after F (11.2 ± 0.2 vs. 10.2 ± 0.3, mmol·L^−1^, *P* < 0.05) than lower-performing competitors.

**Conclusion:**

This study provides novel insights into physiological demands of an entire sprint cross-country skiing competition, which involves repeated 3-min high-intensity efforts interspersed with > 2 h (25 km) of low- to moderate-intensity exercise.

## Introduction

Sprint cross-country (XC) skiing involves approximately 3-min high-intensity efforts (covering a distance of around 1.3–1.8-km) separated by recovery periods lasting between 15 and 120 min (FIS 2022). The individual competition format includes a qualifying time trial (TT), followed by three knockout heats (quarterfinals [QF], semifinals [SF], and final [F]). These repeated high-intensity efforts interspersed with periods of passive recovery and low- to moderate-intensity exercise are unique to sprint XC skiing and create specific competitive demands that differ from most comparable endurance sport events (Hébert-Losier et al. 2017).

While the TT often requires high individual effort from start to finish to qualify, the heats are more influenced by tactics and positioning. Therefore, the speeds at which the heats are performed in sprint XC skiing can be either higher (Andersson et al. 2019), lower (Haugnes et al. 2022; Stöggl et al. 2007), or the same (Mikkola et al. 2010; Vesterinen et al. 2009) compared to the individual TT, resulting in notable differences in speed profiles and pacing strategies (Haugnes et al. 2022). In this context, a “fast-start” or “all-out” pacing strategy appears to be most optimal for improving TT performance (Haugnes et al. 2019; Losnegard et al. 2023). However, corresponding pacing strategies in the subsequent heats are less examined.

The average exercise intensity in sprint XC skiing is reported to be around 110–120% of peak oxygen uptake (VO_2peak_), with an anaerobic energy contribution of approximately 20–25% across three to four repeated efforts in laboratory settings (Losnegard et al. 2012; McGawley and Holmberg 2014; McGawley et al. 2022; Vesterinen et al. 2009) However, the oxygen demand can be considerably higher than the skier’s VO_2peak_ in uphill sections (Andersson et al. 2016, 2017; Sandbakk et al. 2011a, b). These supra-maximal intensities lead to oxygen deficits and require the ability to recover in subsequent downhill sections (Losnegard 2019). It has been suggested that higher-level skiers have a more rapid recovery in the transition between exercise intensities in a simulated competition compared to lower-level skiers (Björklund et al. 2011). Moreover, sprint XC skiing requires the ability to recover between ~ 3 min high-intensity efforts and maintain high physiological effort and performance throughout the entire competition day (Andersson et al. 2016; Losnegard et al. 2015; McGawley et al. 2022). Skiers with higher aerobic capacities and thereby faster blood lactate concentration [La-] clearance between repeated efforts are believed to be better at sustaining their performance (Losnegard et al. 2015), and world-class skiers have demonstrated higher [La-] clearance than national-class sprint skiers (Sandbakk et al. 2011a, b).

Most research with relevance for sprint XC skiing has focused on either the individual TT or repeated efforts using standardized laboratory-based designs (Hébert-Losier et al. 2017). In comparison, comprehensive analysis the performance, physiological and perceptual responses and associated competitive demands of an entire on-snow sprint competition day, which typically lasts around 3–4 h and includes warm-up, recovery between heats (active and passive), and cool-down, have not yet been thoroughly investigated. Moreover, it would be relevant to understand how these features differ between skiers of different performance levels.

Consequently, the primary aim of this study was to investigate performance, physiological, and perceptual responses throughout an entire sprint XC skiing competition in the skating style. Our secondary aim was to compare these responses between the highest and lowest performing skiers. We hypothesized that the best-performing skiers, with higher aerobic capacities, would demonstrate a faster recovery rate, enabling them to sustain elevated levels of physiological and perceptual effort throughout the repeated sprint efforts in the competition.

## Methods

### Participants

Eighteen national-level male junior skiers volunteered to participate in the study. The group had the following mean ± standard deviation (SD) characteristics: age, 18.3 ± 0.7 years; body mass, 74.3 ± 7.9 kg; body height, 181.6 ± 5.4 cm; VO_2peak_ roller-ski skating in the G3 sub-technique, 67.6 ± 5.7 mL·min^−1^·kg^−1^. The study followed the institutional requirements and approval for data security and handling was obtained from the Norwegian Center for Research Data. All participants provided written consent in accordance with the Declaration of Helsinki. Parental consent was obtained for participants aged < 18 years.

### Design

The study involved a simulated on-snow sprint skating competition held in mid-December 2021. The competition was designed to replicate a “real-world” competition comprising an individual qualification TT and subsequent heats (QF, SF, and F). However, a promotion-relegation system was used instead of the regular knockout system to ensure that each participant completed all heats, thus increasing the statistical power of the analyses. Throughout the entire competition day, including warm-up, active and passive recovery between heats, and cool-down, the participants were continuously monitored using heart rate (HR) monitors and global navigation satellite system (GNSS) devices. Perceived “readiness” (RED, 1–10), rating of perceived exertion (RPE, 6–20), and [La-] were assessed at standardized time points. Participants were further divided into high- (HIGH, *n* = 6) and low-performing (LOW, *n* = 6) skiers based on their final rank in the competition for comparisons between performance levels.

### Test protocols and measurements

**Fig. 1:**
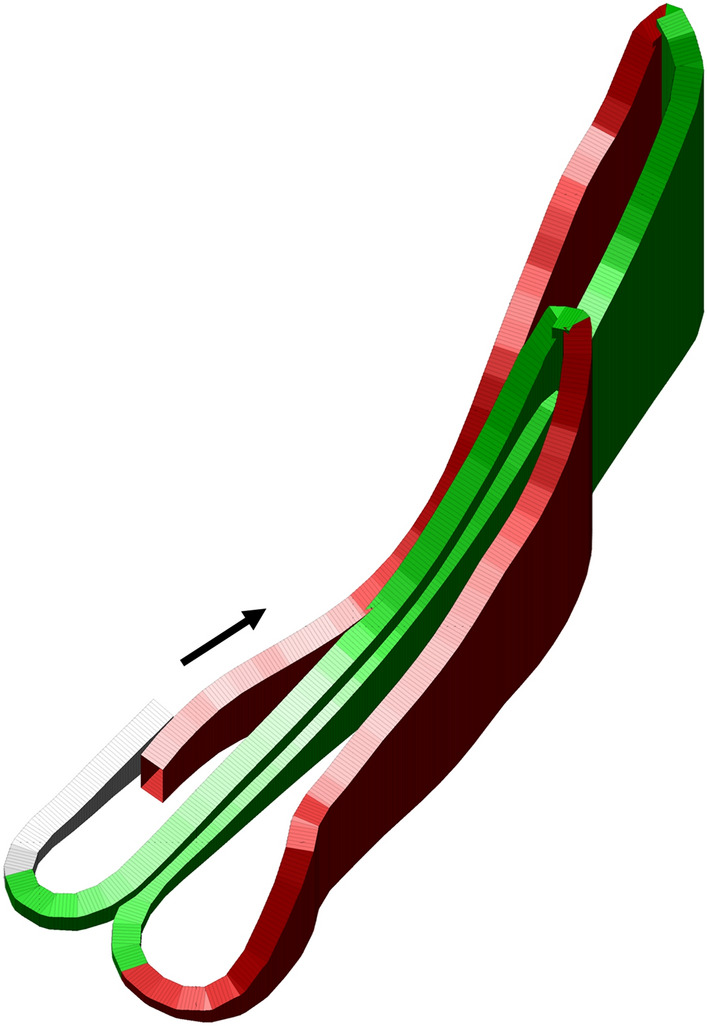
Three-dimensional profile of the course used in the simulated sprint cross-country skiing skating competition divided into five different terrain sections (S1-5). Uphill sections are displayed in red, flat sections in gray, and downhill sections in green

The competition took place in a 1310 m international FIS-regulated course (Fig. 1), which included a combination of artificial and natural snow. The weather conditions remained stable throughout the competition day with the following mean (range) values: ambient air temperature, − 2.1 °C (− 1.6 to − 2.4 °C); snow temperature, − 2.8 °C (− 1.7 to − 3.7 °C); relative humidity, 78% (77–79%).

**Fig. 2:**
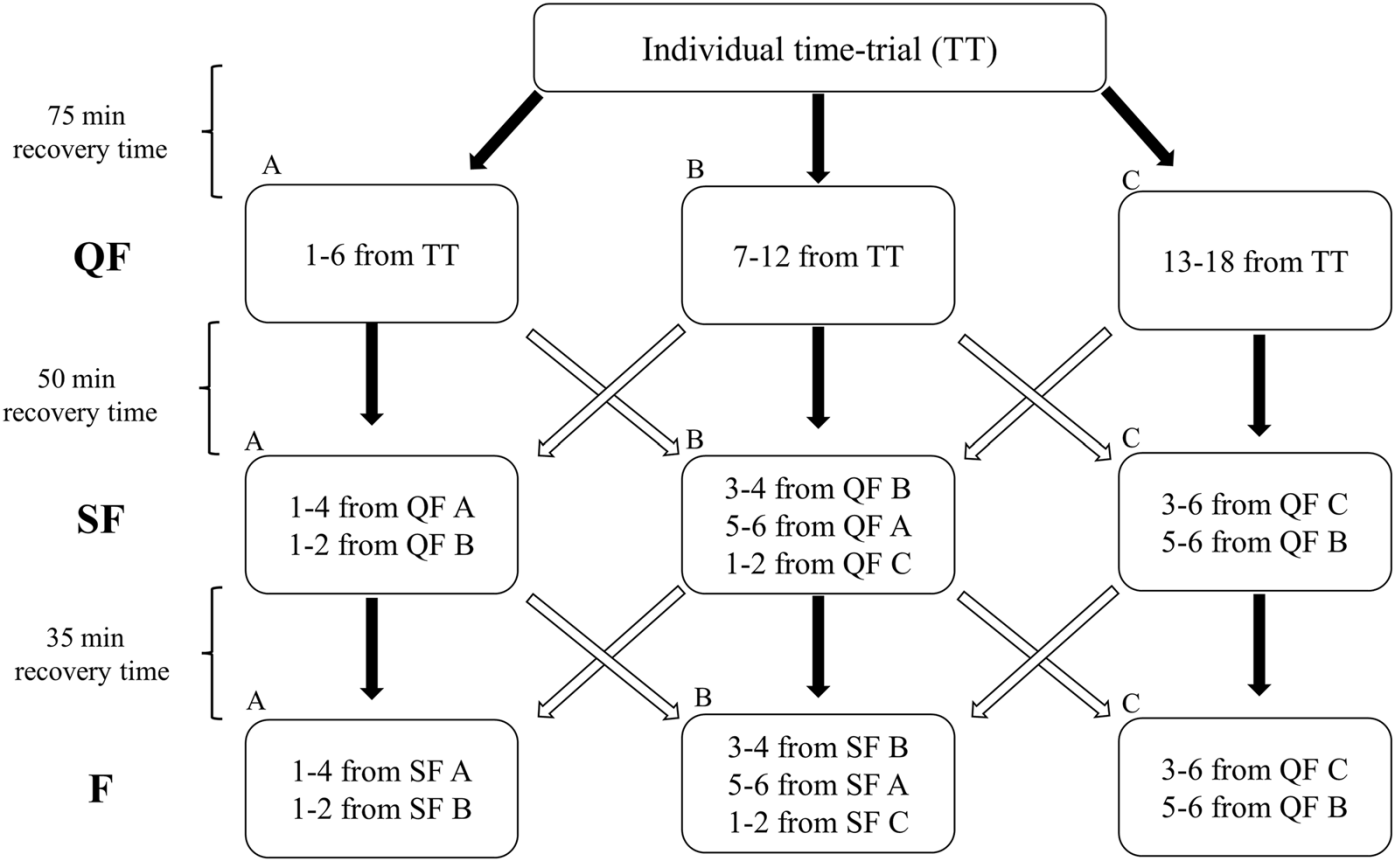
Complete overview of the promotion and relegation system used in the simulated sprint cross-country skiing skating competition. TT indicates individual time trial; QF, quarterfinals; SF, semifinals; F, final

Participants were instructed to engage in standardized low-intensity training the day before the competition and to follow the same nutritional strategies as in a “real-world” competition, and avoiding caffeinated beverages to minimize ergogenic influences. They were also instructed to follow their self-selected procedures throughout the entire competition day, including warm-up, recovery between heats, and cool-down. On the morning of the competition day, participants were equipped with HR monitors and 1 Hz GNSS Garmin Forerunner 920XT/935 watches with electrode belts (Garmin Ltd., Olathe, USA). They had approximately 1 h available for warm-up and subsequently, participants were equipped with combined 10 Hz GNSS and inertial measurement units (IMU) (Optimeye S5, Catapult Sports, Melbourne, Australia) worn in a customized bib on the torso. These devices were put on and taken off between the TT and heats. The TT was performed with 1-min starting intervals, and participants' ranks were used to assign them to three subsequent QF heats (A-B-C heats). Instead of the regular knock-out system used in official sprint XC skiing competitions (FIS 2022), the study employed a promotion and relegation system, where the two fastest and slowest participants in each heat were promoted and relegated, respectively (see Fig. 2 for a complete overview of the promotion-relegation system used). Although all participants “qualified” to QF and finished all subsequent heats, participants were instructed by their coaches to give full effort throughout the entire competition day. The recovery times between TT, QF, SF, and F were set to 75, 50, and 35 min, respectively, following the FIS competition rules (FIS 2022). However, time between TT and QF was shorter (normally 1.5–2.0 h), and the time between SF and F was longer (normally 15–20 min) than the official FIS competition rules due to logistical considerations. Approximately 5 min before TT and all heats, [La−] was measured from the participants' fingertip, along with asking for RED. Approximately 2 min after each effort, [La−] was assessed again together with RPE. Participants' maximal heart rate (HR_max_) was determined by taking their peak heart rate (HR_peak_) from an incremental test treadmill roller-ski skating (G3 sub-technique) in the laboratory and adding 5 bpm (Ingjer 1991). Time spent > 60% of HR_max_ and time spent > 85% of HR_max_ were calculated for the entire competition day. Furthermore, HR_peak_ and mean heart rate (HR_mean_) in different efforts/parts of the competition were calculated for each participant.

The data of each participant was adopted to the defined course and elevation profile (Fig. 1) by aligning each participant 10 Hz GNSS track with points along the reference course. The methodology used for this adjustment has previously been described by (Sandbakk et al. 2016) and both GNSS sensors used in the study have been validated against a differential GNSS sensor (Gløersen et al. 2018). The course was further divided into uphill, flat, and downhill terrain sections, comprising five different sections: S1, uphill; S2, downhill; S3, uphill; S4, downhill; S5, flat (final sprint). Classification of different terrain sections followed the guidelines outlined in the FIS homologation manual for XC skiing courses (FIS 2022). A section boundary was defined where a change between positive and negative gradient in the course occurred. Uphill sections were defined as those with a climb of more than 10 m and a gradient of more than 6%, while downhill sections were defined as those with a descent of more than 10 m and a negative gradient of more than 6%. Remaining sections were classified as flat terrain sections. [La-] was taken from the participants fingertip using a Lactate Pro 2 sensor (Arkray Europe B.V, Amstelveen, Netherlands) while the 6–20 Borg scale (Borg 1970) was used to determine RPE. Perceived RED was reported on a scale ranging from 1 (poor) to 10 (excellent). Participants used their own ski equipment, including poles, boots, and skis optimized according to their individual preferences. Additionally, participants were instructed to prepare their skis with the same fluorine-free glide wax before the competition.

### Statistical analysis

Data are presented as mean ± SD. A one-way repeated measures ANOVA was used to compare performance, physiological, and perceptual responses across different parts of the competition. In cases of any global differences, Fisher LSD post hoc analyses were conducted to determine the specific locations of the differences. Due to the small number of participants in each group, the non-parametric Mann–Whitney *U* test was used to assess statistically significant differences between high- (A-final) and low-performing (C-final) skiers. The level of statistical significance was set at alpha < 0.05 and all statistical analyses were carried out using SPSS 26.0 (SPSS Inc, Chicago, IL, USA).

## Results

Total time and distance covered during the entire competition day (including warm-up, passive and active recovery between heats, and cool-down) were 03:30 ± 00:06 h and 25.2 ± 2.9 km, respectively. Participants spent a total of 02:19 ± 00:27 h > 60% of HR_max_ and 00:16 ± 00:04 h > 85% of HR_max_.

**Fig. 3:**
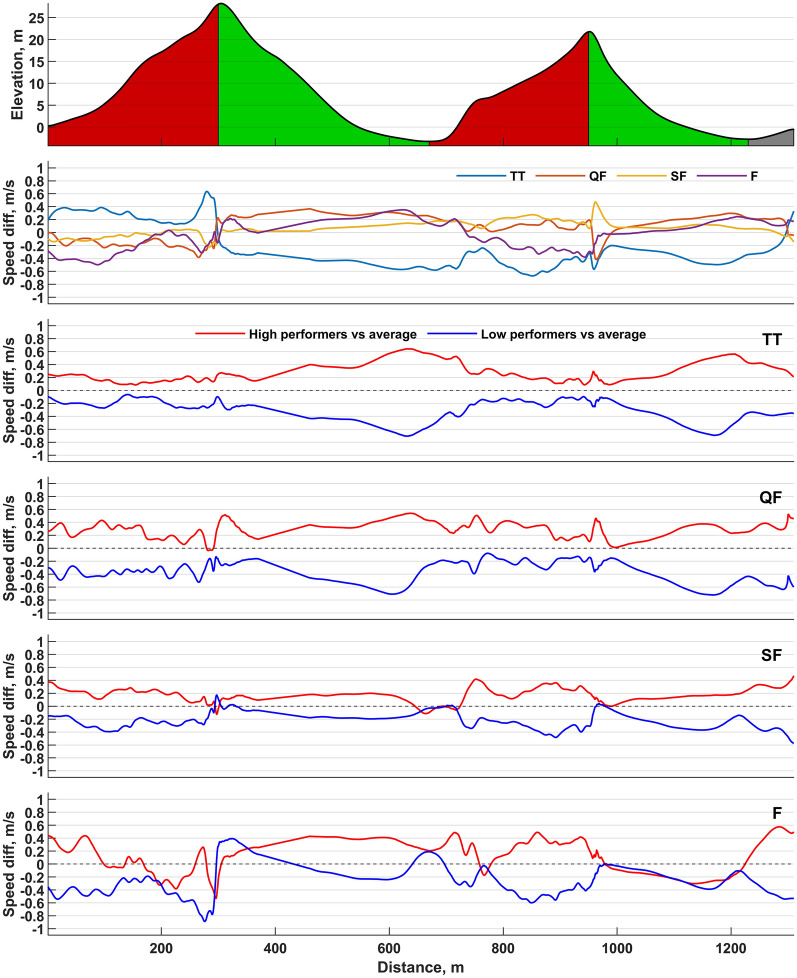
Speed differences between the individual time trial and subsequent heats as well as between high- and low-performing skiers compared to average speed during a simulated sprint cross-country skiing skating competition. Uphill sections are displayed in red, flat sections in gray, and downhill sections in green. TT indicates individual time trial; QF, quarterfinals; SF, semifinals; F, final

Participants were faster in SF than in TT, QF, and F (all *P* < 0.05, Table 1, Fig. 3). During TT, participants were faster in S1 compared to all heats (*P* < 0.001), while they were slower in S2 during TT compared to all heats (all *P* < 0.001). Participants were slower in S3 during TT than in the heats (*P* < 0.001) and faster in SF than in F (*P* = 0.002). Participants were also slower in S4 during TT compared to all heats (*P* < 0.001), while no time differences in S5 were found between TT and heats. HIGH were faster both overall and within different sections compared to LOW in TT and all heats (all *P* < 0.05, Fig. 3).

**Table 1:** Total and section-specific times and speeds in the individual time trial and subsequent heats of a simulated sprint cross-country skiing skating competition (*n* = 18)

Variables	TT	QF	SF	*F*	Avg	*P
Total time (s)	176.4 ± 6.9	175.3 ± 8.1	172.7 ± 7.7	179.4 ± 12.5	176.1 ± 7.1	0.008
Speed (m/s)	7.41 ± 0.30	7.44 ± 0.36	7.58 ± 0.34	7.42 ± 0.32	7.50 ± 0.29	0.005
Section 1 (s)	57.9 ± 3.4	65.8 ± 5.3	62.6 ± 5.3	65.4 ± 6.0	62.5 ± 3.6	< 0.001
Speed (m/s)	5.2 ± 0.3	4.6 ± 0.4	4.8 ± 0.4	4.6 ± 0.4	4.8 ± 0.3	< 0.001
Section 2 (s)	31.4 ± 1.0	29.9 ± 1.3	29.9 ± 0.7	29.8 ± 0.7	30.0 ± 0.6	< 0.001
Speed (m/s)	11.8 ± 0.4	12.5 ± 0.5	12.4 ± 0.3	12.4 ± 0.3	12.3 ± 0.2	< 0.001
Section 3 (s)	53.6 ± 2.7	48.2 ± 2.4	47.8 ± 2.5	49.0 ± 3.0	49.4 ± 2.3	< 0.001
Speed (m/s)	5.2 ± 0.3	5.8 ± 0.3	5.9 ± 0.3	5.7 ± 0.4	5.7 ± 0.3	< 0.001
Section 4 (s)	24.8 ± 0.9	23.7 ± 0.9	23.5 ± 0.6	23.4 ± 0.7	23.8 ± 0.7	< 0.001
Speed (m/s)	11.3 ± 0.4	11.8 ± 0.5	11.9 ± 0.3	12.0 ± 0.4	11.8 ± 0.3	< 0.001
Section 5 (s)	9.6 ± 0.5	9.3 ± 0.7	9.5 ± 0.8	9.1 ± 0.5	9.3 ± 0.5	0.053
Speed (m/s)	8.4 ± 0.4	8.7 ± 0.6	8.5 ± 0.6	8.8 ± 0.4	8.6 ± 0.4	0.057

Data are presented as mean ± standard deviation

*One-way repeated-measures ANOVA

*TT* indicates time trial, *QF* quarterfinal, *SF* semifinal, *F* final

**Fig. 4:**
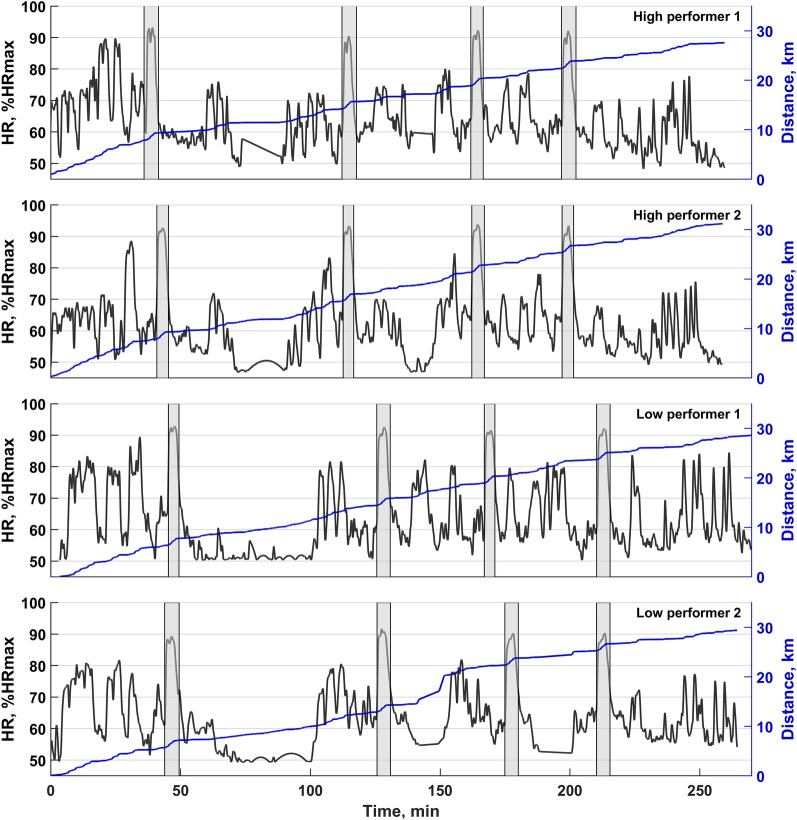
Individual heart-rate profiles and distance covered during an entire sprint cross-country skiing skating competition day (including warm-up and recovery periods) for two high- and low-performing skiers. HR indicates heart rate; HR_max_, maximal heart rate

HR_mean_ decreased from TT to F (*P* = 0.001) and HR_peak_ decreased from TT to SF and F (both *P* < 0.05, Table 2). There were no differences in HR responses between HIGH and LOW in TT and heats, although there was a tendency for lower HR_mean_ in F for LOW compared to HIGH (85.2 ± 2.4 vs. 87.8 ± 1.4% of HR_max_, *P* = 0.069). Examples of individual HR profiles for two high- and low-performing skiers during the entire sprint competition day are displayed in Fig. 4.

**Table 2:** Physiological and perceptual responses in the individual time trial and subsequent heats of a simulated sprint cross-country skiing skating competition (*n* = 18)

Variables	TT	QF	SF	*F*	Avg	*P
HR_mean_ (%HR_max_)	89.3 ± 2.0	88.7 ± 2.1	87.9 ± 3.7	86.9 ± 3.0	88.2 ± 2.4	0.004
HR_peak_ (%HR_max_)	92.3 ± 1.9	91.9 ± 2.0	91.3 ± 2.5	90.2 ± 2.8	91.4 ± 2.1	< 0.001
PRE [La^−^] (mmol·L^−1^)	2.9 ± 1.2	3.2 ± 2.0	2.5 ± 1.3	4.6 ± 2.0	3.2 ± 1.1	0.006
POST [La^−^] (mmol·L^−1^)	9.9 ± 1.6	9.1 ± 1.8	8.7 ± 1.7	10.8 ± 1.4	9.6 ± 1.0	< 0.001
RED (1–10)	8.0 ± 2.0	8.0 ± 1.0	7.0 ± 2.0	6.5 ± 2.0	7.5 ± 1.5	0.002
RPE (6–20)	18.0 ± 1.0	15.0 ± 2.0	16.0 ± 2.0	17.0 ± 3.0	16.5 ± 1.5	< 0.001

Data are presented as mean ± standard deviation

*One-way repeated-measures ANOVA

*TT* indicates time trial, *QF* quarterfinal, *SF* semifinal, *F* final, *HR*_*mean*_ mean heart rate, *HR*_*peak*_ peak heart rate, *HR*_*max*_ maximal heart rate, *[La*^*−*^*]* blood lactate consentrations, *RED* perceived readiness, *RPE* rating of perceived exertion

**Fig. 5:**
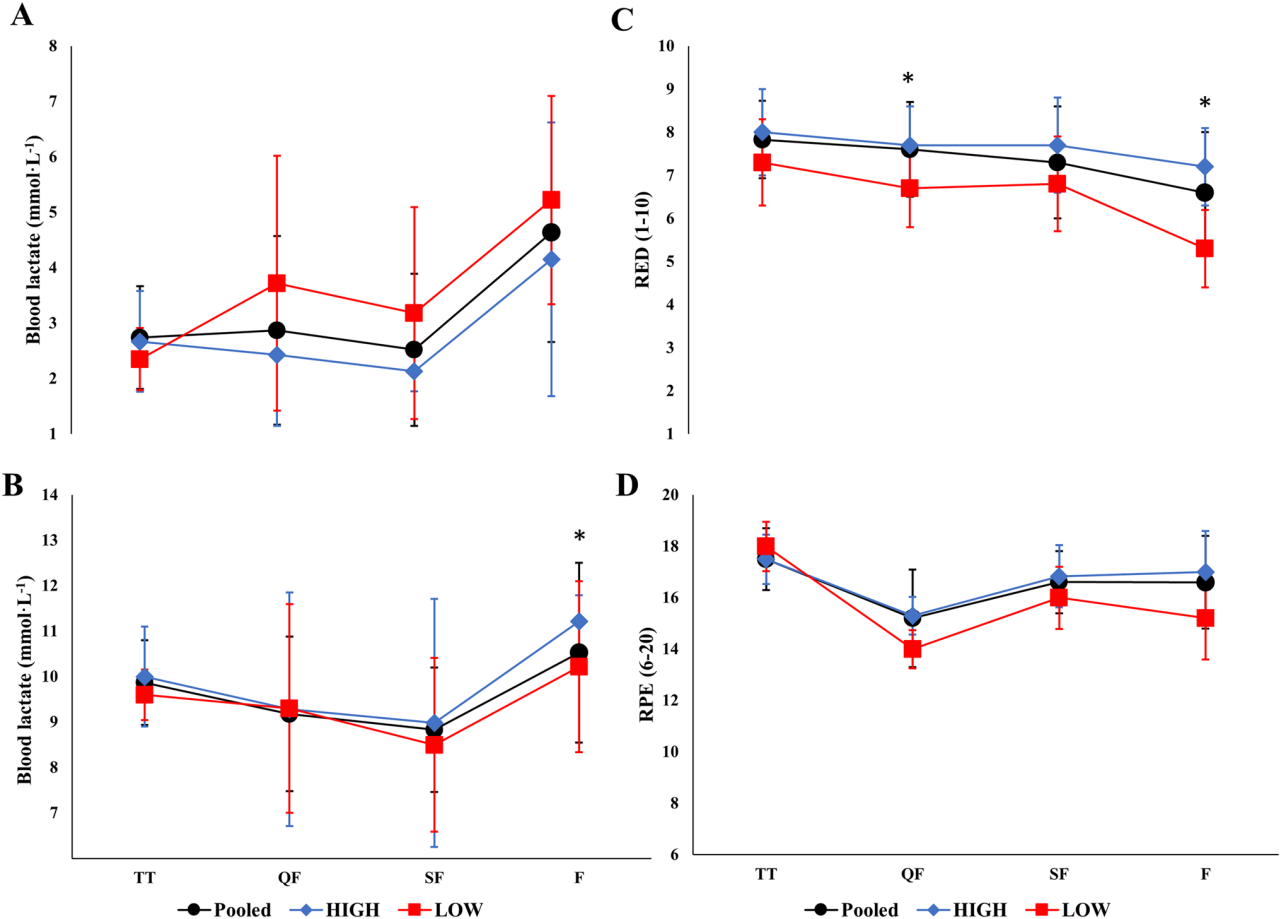
Blood lactate concentrations before (**A**) and after (**B**), perceived readiness before (**C**), and rating of perceived exertion after (**D**) the individual time trial and subsequent heats of a simulated sprint cross-country skiing skating competition for all participants as well as for high- and low-performing skiers. *TT* indicates individual time trial, *QF* quarterfinals, *SF* semifinals, *F* final, *RED* perceived readiness, *RPE* rating of perceived exertion, *HIGH* high performing, *LOW* low performing. *Significant difference between HIGH and LOW (*P* < 0.05)

[La^−^] was highest before F compared to TT and the other heats (all *P* < 0.05, Table 2, Fig. 5) and highest after F compared to QF and SF (all *P* < 0.01). [La^−^] clearance was lower between SF and F than between TT and QF and between QF and SF (all *P* < 0.01). RED was lowest before F compared to TT and other heats (all *P* < 0.05) and RPE was highest after TT compared to all heats (all *P* < 0.05, Table 2). HIGH demonstrated higher [La^−^] after F compared to LOW (11.2 ± 0.2 vs. 10.2 ± 0.3 mmol·L^−1^, *P* = 0.043), with no other significant differences in [La^−^] or [La^−^] clearance found between the two performance groups. HIGH demonstrated greater RED compared to LOW both before QF and F (7.7 ± 1.0 vs. 6.7 ± 1.0 and 7.2 ± 0.9 vs. 5.3 ± 1.2, both *P* < 0.05, Fig. 5), while RPE after TT and heats did not differ significantly between groups.

Participants’ time and distance covered as well as physiological responses during warm-up and recovery periods are shown in Table 3. Both HR_mean_ and HR_peak_ during warm-up were higher than during the recovery periods (all *P* < 0.001). Participants spent relatively more time both > 60% of HR_max_ and > 85% of HR_max_ during warm-up compared to the recovery periods. There were no differences between HIGH and LOW in time used and HR responses during warm-up and recovery periods, although HIGH covered more distance during warm-up compared to LOW (7.6 ± 0.4 vs. 6.3 ± 0.7 km, *P* = 0.012).

**Table 3:** Time and distance covered in connection with warm-up and recovery periods between the individual time trial and subsequent heats of a simulated sprint cross-country skiing skating competition (*n* = 18)

	Warm-up	Recovery 1	Recovery 2	Recovery 3	*P
Time (min)	44.5 ± 10.7	70.2 ± 5.5	46.5 ± 5.1	32.5 ± 5.3	< 0.001
Distance (km)	7.1 ± 1.1	6.0 ± 1.5	4.2 ± 1.3	2.3 ± 0.9	< 0.001
HR_mean_ (%HR_max_)	69.5 ± 3.5	59.1 ± 3.8	61.9 ± 2.6	59.3 ± 4.0	< 0.001
HR_peak_ (%HR_max_)	89.8 ± 2.7	83.9 ± 3.4	83.2 ± 2.8	78.0 ± 5.9	< 0.001
Time > 60% of HR_max_ (%)	76.6 ± 7.6	42.4 ± 15.6	65.2 ± 18.2	18.4 ± 6.7	< 0.001
Time > 85% of HR_max_ (%)	6.2 ± 5.5	0.5 ± 1.2	0.0 ± 0.1	0.0 ± 0.1	< 0.001
[La^−^] clearance (mmol·L^−1^)	Na	6.7 ± 2.3	6.7 ± 1.9	4.1 ± 2.1	0.001

Data are presented as mean ± standard deviation

*One-way repeated-measures ANOVA

*Recovery 1* indicates period between the individual time trial and quarterfinals, *Recovery 2* period between quarterfinals and semifinals, *Recovery 3* period between semifinals and finals, *HR*_*mean*_ mean heart rate, *HR*_*peak*_ peak heart rate, *HR*_*max*_ maximal heart rate, *[La*^*−*^*]* blood lactate concentrations, *Na* not available

## Discussion

This study investigated performance, physiological, and perceptual responses during an entire sprint XC skiing competition in the skating style and compared these features between the highest- and lowest-performing skiers. The main findings were: (1) the entire competition day encompassed approximately 25 km of skiing and lasted around 3.5 h, including approximately 15 min of high-intensity exercise interspersed with around 2 h of low- to moderate-intensity exercise; (2) SF was fastest, while F was slower than TT, exhibiting considerable differences in their respective speed profiles; (3) both HR_mean_ and HR_peak_ decreased from TT to F, with the highest levels of [La-] observed before and after F, and additionally, RED was lowest before F and RPE highest after TT; (4) the best-performing skiers reported higher RED before QF and F, and exhibited higher levels of [La-] after F compared to their lower-performing competitors.

This study provides a comprehensive analysis and novel insight into the physiological and perceptual demands of an entire sprint XC skiing competition day, including warm-up and recovery periods. In addition to four repeated 3-min high-intensity efforts, the simulated competition encompassed a total duration of around 3.5 h, including 2 h of low- to moderate-intensity exercise and 25 km of skiing. These specific competitive demands distinguish sprint XC skiing from most comparable endurance sport events and should be considered when designing appropriate training programs for sprint XC skiers.

Participants demonstrated higher speeds in SF compared to TT, QF, and F, while F was slower than TT. These patterns align with previous research, which has indicated that heats are performed at either higher (Andersson et al. 2019), lower (Haugnes et al. 2022; Stöggl et al. 2007), or the same speeds (Mikkola et al. 2010; Vesterinen et al. 2009) as compared to TT, depending on various factors such as tactics and the composition of study groups. Skiing in a pack during heats offers advantages such as reduced air drag and snow friction (Seeberg et al. 2022a, b), as well as potential time gains due to skiers' varying strengths on different sections of the course. It is also logical that SF requires higher speeds to increase the chances of qualifying for F and achieving a high overall ranking, while F involves slower speeds and greater tactical considerations. Consequently, significant disparities in speed profiles seem to arise between the individual TT and different heats in sprint XC skiing.

Specifically, participants exhibited higher speeds in the first uphill section (S1), but lower speeds in S2–4, and particularly S3 (uphill), during TT compared to the heats. This finding aligns with a recent study by Haugnes et al. (2022) who investigated a “real-world” classical sprint competition among elite XC skiers, highlighting considerable differences in the speed profiles (i.e., pacing strategies) adopted in the individual TT compared to the subsequent heats. These differences can be attributed to lower speeds and greater emphasis on positioning in the initial part of the heats, followed by higher speeds to outpace competitors in the latter part. In contrast, TT is characterized by higher effort already from start and an optimal distribution of speed and metabolic energy from start to finish is required. Interestingly, a recent study by Losnegard et al. (2023) investigating a comparable group of female junior skiers found that pacing strategy in TT was dependent on the skier’s performance level, with higher-performing skiers better able to tolerate a “fast-start” pattern. Collectively, these findings emphasize the importance of mastering different pacing strategies in sprint XC skiing due to considerable differences in speed profiles between TT and subsequent heats. While the individual TT involves a “fast-start” or “all-out” pacing strategy, the subsequent heats typically feature a more “conservative start” with gradually increasing speeds, where positioning and tactics play crucial roles in determining the final outcome. Moreover, the highest-performing skiers were faster in all terrain sections compared to their lower-performing competitors, although the most pronounced relative speed differences were found in the downhill sections. This could in part be attributed to higher speeds over hilltops leading to better performance in subsequent downhill sections (Seeberg et al. 2022a, b) among the highest-performing skiers, while variations between skis cannot be ruled out as measures of friction were not included in the study.

Both HR_mean_ and HR_peak_ decreased throughout the competition, with significantly lower values observed in F compared to TT. These findings differ from laboratory-based studies investigating repeated efforts in sprint XC skiing (Stöggl et al. 2007; Vesterinen et al. 2009), but are consistent with those reported by Andersson et al. (2019), who employed a similar design in the classical style. While the observed reductions in HR can partly be attributed to the lower speeds observed in F, the highest [La-] values were observed after F. These physiological responses were accompanied by a gradual decline in perceived “readiness” before each effort from TT to SF and F. However, the best-performing skiers tended to have higher mean HR in F and demonstrated greater “readiness” both before QF and F compared to their lower-performing competitors. Conversely, the highest levels of RPE were reported after TT which can be attributed to the “fast-start” pattern and the requirement for high individual effort already from the start. These findings align with the study by Losnegard et al. (2023), who demonstrated a higher level of “discomfort” associated with a “fast-start” strategy in TT. However, no differences in RPE between the two performance groups were observed, although the reduced “readiness” might also reflect a decrease in motivation among the lower-performing skiers throughout the competition day. Overall, levels of “readiness” and physiological effort decreases as the competition progresses and recovery times between heats shorten. However, these factors also appear to differentiate high-performing skiers from lower-performing ones.

Peak [La-] values observed after each effort were on average 9.6 mmol·L^−1^ and consistent with the values reported in previous studies (Andersson et al. 2019; Losnegard et al. 2015; McGawley et al. 2022; Stöggl et al. 2007; Zory et al. 2006). Furthermore, the gradual increase in [La-], with the highest values found after F, aligns with the study by Andersson et al. (2019). Additionally, participants exhibited mean [La-] values of > 2.5 mmol·L^−1^ before TT and reduced [La-] to the same levels before the subsequent heats, which are in line with the values reported in comparable studies (Losnegard et al. 2015; McGawley et al. 2022; Vesterinen et al. 2009). Similar to the findings of Vesterinen et al. (2009), higher levels of [La-] were observed before F due to lower [La-] clearance in the shorter recovery period between SF and F. These findings are further supported by McGawley et al. (2022) who demonstrated lower [La-] clearance and impaired performance with shorter compared to longer recovery periods, simulating the scenario of different heat selections in sprint XC skiing. However, even though there were numerical differences in [La-] before the heats (~ 1 mmol·L^−1^) and [La-] clearance in the recovery periods (~ 1.5 mmol·L^−1^) between high- and lower-performing skiers, these differences did not reach statistical significance. The rate of [La-] clearance has previously been associated with the ability to sustain performance in sprint XC skiing(Losnegard et al. 2015) and suggested it to be a feature separating skiers of different performance levels (Björklund et al. 2011; Sandbakk et al. 2011a, b). Although [La-] does not represent a valid physiological measure of either anaerobic energy contribution, muscle fatigue or recovery (Allen et al. 2008), the ability to clear and (re)produce [La-] is associated with such mechanisms.

The higher mean and peak HR values, as well as more time spent at higher exercise intensities during warm-up compared to the recovery periods, were as expected, and likely reflects the participants’ self-selected warm-up and recovery strategies. While warm-up aims to physically and mentally prepare skiers for the first high-intensity effort (i.e., individual TT), the recovery periods are more characterized by “recovery” (both passive and active) with the aim of maintaining high physiological function and performance in the subsequent heats (Losnegard et al. 2015). The recovery periods between heats therefore involves the complexity of both recovering and optimally preparing (“warming up”) for the next heat.

## Conclusion and practical applications

This study provides novel insights into the physiological and perceptual demands of an entire sprint XC skiing skating competition. In addition to performing four repeated ~ 3-min bouts of high-intensity exercise across varying terrains, the competition day also entails approximately 2 h (25 km) of low- to moderate-intensity skiing over a total duration of 3.5 h. The ability to consistently repeat exceptional performance and sustain heightened physiological and perceptual effort throughout the competition distinguishes the highest-performing skiers from their lower-performing competitors. However, it should be noted in the interpretation of the findings that this was a simulated competition including a promotion and relegation system with some differences in recovery times compared to an official sprint XC skiing competition. Altogether, these unique competitive demands are exclusive to sprint XC skiing and distinguish it from most other endurance sports. Therefore, it is imperative to meticulously consider all these demands when designing appropriate training programs for sprint XC skiers.

## Data Availability

The datasets are available from the corresponding author on reasonable request.

## Abbreviations

F: Final
GNSS: Global navigation satellite system
HIGH: High performers
HR: Heart rate
HR_mean_: Mean heart rate
HR_max_: Maximal heart rate
HR_peak_: Peak heart rate
IMU: Inertial measurement unit
[La-]: Blood lactate concentration
LOW: Low performers
QF: Quarterfinals
RED: Perceived readiness
RPE: Rating of perceived exertion
SD: Standard deviation
SF: Semifinals
TT: Time trial
VO_2peak_: Peak oxygen uptake
XC: Cross-country
